# Comorbidity and intercurrent diseases in geriatric stroke rehabilitation: a multicentre observational study in skilled nursing facilities

**DOI:** 10.1007/s41999-018-0043-5

**Published:** 2018-03-13

**Authors:** Anouk D. Kabboord, Monica Van Eijk, Bianca I. Buijck, Raymond T. C. M. Koopmans, Romke van Balen, Wilco P. Achterberg

**Affiliations:** 1 Department of Public Health and Primary Care, Leiden University Medical Center, Hippocratespad 21, Postbus 9600, 2300 RC Leiden, The Netherlands; 2 Department of Neurology, Erasmus MC University Medical Center, ‘s-Gravendijkwal 230, Rotterdam, The Netherlands; 3 Department of Primary and Community Care, Centre for Family Medicine, Geriatric Care and Public Health, Radboud University Nijmegen Medical Centre, Nijmegen, The Netherlands; 4 “Joachim en Anna”, Centre for Specialized Geriatric Care, Nijmegen, The Netherlands

**Keywords:** Geriatric rehabilitation, Comorbidity, Functional impairments, Intercurrent diseases, Stroke

## Abstract

**Background:**

Older patients often have multiple comorbidities and are susceptible to develop intercurrent diseases during rehabilitation. This study investigates intercurrent diseases and associated factors in patients undergoing geriatric stroke rehabilitation, focussing on pre-existing comorbid conditions, overall comorbidity and baseline functional status.

**Materials and methods:**

This multicentre prospective cohort study included 15 skilled nursing facilities. Data were collected at baseline and at discharge. The primary outcome measures were presence and number of intercurrent diseases. Furthermore, their impact on change in rehabilitation goals or length of stay was examined. Comorbidity was assessed with the Charlson index, and functional status with the Barthel index (BI).

**Results:**

Of the 175 included patients, 51% developed an intercurrent disease. A lower baseline BI, a higher Charlson index, presence of diabetes mellitus (DM) and kidney disease were related to the occurrence of an intercurrent disease (*p* < 0.05). Moreover, a lower BI, a higher Charlson index, and particularly the presence of DM were independently associated. If both comorbidity and a lower baseline functional status were present, the odds ratio (95% CI) of developing intercurrent diseases was 6.70 [2.33–19.2], compared to 1.73 [0.52–5.72] (comorbidity only) and 1.62 [0.53–4.94] (only BI ≤ 14).

**Conclusions:**

On admission, functional impairments and comorbidity, particularly diabetes, independently contribute to developing intercurrent diseases during geriatric stroke rehabilitation. Therefore, routine evaluation of comorbidity integrated with functional status at the start of rehabilitation is essential to identify patients at risk. Finally, particular attention should be paid to patients with DM to prevent intercurrent diseases and support optimal functional recovery .

**Electronic supplementary material:**

The online version of this article (10.1007/s41999-018-0043-5) contains supplementary material, which is available to authorized users.

## Introduction

Following acute hospitalisation, rehabilitation helps patients to regain functional independency that enables them to be discharged home. However, during hospitalisation, the risk of functional decline and complications is particularly increased in older patients [1]. In the Netherlands, about one-third of all stroke patients are referred to a skilled nursing facility (SNF) that provides geriatric rehabilitation. These patients are usually relatively older, have a longer length of stay (LoS) in the acute hospital, and have more complex problems (Supplement material Appendix A) [2]. Also, during inpatient rehabilitation, intercurrent diseases may occur that interfere with therapy and could negatively impact rehabilitation outcome [3, 4].

Studies investigating complications during inpatient stroke rehabilitation found that 30–96% of the patients developed complications; this wide range could be due to different definitions of a complication and the methods of measurement [5–12]. The present study investigates intercurrent diseases, i.e. *any disease that occurs during the progress of another disease,* during rehabilitation. Factors related to intercurrent diseases can include age, gender [9, 13], time interval between stroke and rehabilitation [7, 10, 12], severe stroke [7, 11] or functional impairment [6, 9, 12, 13] and comorbidity [5–7, 13, 14], although it is unknown which specific comorbidities are related. Particularly, older patients are at risk of functional decline and often have multiple comorbidities. However, few studies have investigated associations with intercurrent diseases in the older, vulnerable group of patients receiving geriatric stroke rehabilitation [5, 13]. Furthermore, intercurrent diseases may impede successful functional recovery [5, 14]. Therefore, to better understand the relations between comorbidity, functional impairment and intercurrent diseases, and to identify associated pre-existing comorbid conditions, this study explores: (i) the presence, and number of intercurrent diseases and their impact on older patients admitted to an SNF, recovering after stroke, and (ii) factors associated with the presence and number of intercurrent diseases, focusing on functional status and comorbidity.

## Materials and methods

### Participants

Data were obtained from the Geriatric Rehabilitation in AMPutation and Stroke (GRAMPS) study. Data collection took place between January 2008 and July 2010; details on the study design are already published [15]. A total of 15 SNFs located in the southern part of the Netherlands participated. All stroke patients admitted to one of these SNFs were eligible for inclusion. Patients were excluded if they refused participation, were unable to give informed consent, were critically ill, or were expected to have a stay of ≤ 2 weeks. The medical ethics committee of the region Nijmegen-Arnhem approved the study protocol.

### Outcome measures

For the present study, the outcome measures were: the presence and number of intercurrent diseases that occurred during rehabilitation. Intercurrent diseases were coded using the 10th revision Clinical Modification ICD-10CM. At discharge, the attending physician registered intercurrent diseases that affected the course of the rehabilitation: impact was classified according to (i) whether the disease had prolonged the LoS or (ii) whether the rehabilitation goals needed adjustment. Four categories were formed: (1) no intercurrent disease, (2) ‘No impact’, (3) ‘With impact’, and (4) intercurrent disease that directly caused death.

### Data collection

The participating multidisciplinary teams consisted of a physician [16], a physiotherapist, an occupational therapist, a psychologist, a speech therapist, a dietician and skilled nurses; all received the same instructions regarding performance of the assessments. Data were collected within the first 2 weeks after admission (T0) and at discharge (T1) from the SNF or (at the latest) 1 year after admission, if a patient was still in the SNF at that time.

### Measurements

The following patient characteristics and data were collected: age, gender, home situation, comorbidity, LoS in acute hospital, LoS in the SNF, and discharge destination [5–14, 17]. Functional assessment was performed at baseline and at discharge using the modified Barthel index (BI) to assess activities of daily living (ADL) [18]. Premorbid BI was assessed on admission, using information on the patient’s situation prior to the acute stroke, based on interview and collateral history. Functional recovery was defined in two ways: BI at discharge and ‘relative functional gain’, which was calculated as follows: (BI-discharge minus BI-admission)/(BI-premorbid minus BI-admission) × 100 [19, 20]. Relative functional gain expresses the achieved percentage of potential functional gain.

Pre-existing comorbidity was assessed using the Charlson comorbidity index (Charlson-CI). This index consists of 19 diagnoses and was adjusted for stroke [21–23]. The Charlson-CI was categorised as: 0 (no comorbidity), 1 (single comorbidity) or ≥ 2 (multiple comorbidities), unless otherwise specified. Comorbidities were recorded if present in medical history, e.g. chronic diseases and conditions that required ongoing use of (preventive) medication. Conditions that had completely resolved without any residual symptoms or need for treatment were not noted (e.g. childhood asthma). Finally, if myocardial infarction in the past had led to heart failure, only heart failure was recorded.

### Statistical analysis

Data were processed and analysed using the Statistical Package for Social Science version 23. Means with standard deviations (normal distribution), medians with interquartile ranges (skewed data), or absolute numbers with percentages (categorical data) are reported.

A Chi-Squared test (categorical data), ANOVA or Kruskal–Wallis test, depending on their distribution, were used to detect mean differences in characteristics between the four intercurrent disease categories and to identify comorbid conditions related to the occurrence of intercurrent diseases. A *p* value of ≤ 0.05 was considered statistically significant.

Multivariate analyses were performed using binary logistic regression with the presence of intercurrent diseases and Poisson regression with number of intercurrent diseases as the dependent variable. Rehabilitation LoS (log) was added as the ‘offset’. Factors included in the multivariate model were age and gender. Significant baseline variables (*p* < 0.10) were added as a continuous variable if applicable.

Before performing the analyses, data were tested for the required assumptions, such as multicollinearity, interaction and effect modification. To investigate comorbidity and baseline functional status, separate and combined relations with the presence of intercurrent diseases were analysed. For this purpose, variables were dichotomized. Odds ratios (OR) were calculated with the absence of both factors as reference category [24]. Sensitivity analyses were performed, i.e. with and without deceased patients.

## Results

### Characteristics

Of the 378 eligible patients, 186 were included in the GRAMPS study; the excluded patients did not differ with regard to age, gender or LoS [25]. The present study included 175 patients because 11 patients were lost to follow-up, mainly due to translocation to another SNF (Supplement material Appendices B and C). Table 1 presents the baseline characteristics of the study population, and the intercurrent disease categories. Mean age was 78.8 years and 46% were males. On average, LoS in the acute hospital was 19 days, the premorbid BI was 20, baseline BI was 12, and BI at discharge was 17. LoS in the SNF was 12 weeks, the (average) relative functional gain was 67, and 56% of these patients was discharged home.

**Table 1:** Patient characteristics classified by intercurrent disease (ID) impact category

	Total baseline *n* = 175	ID absent *n* = 86	ID no impact *n* = 22	ID with impact *n* = 46	ID deceased *n* = 16	ID impact unknown *n* = 5
*Variables at baseline*						
Age (years), Mean (SD)	78.8 (8.0)	78.2 (8.3)	78.8 (5.6)	78.9 (8.5)	81.2 (8.4)	82.6 (7.8)
Gender (male), *n* (%)	80 (46)	45 (52)	11 (50)	16 (35)	7 (44)	1 (20)
Charlson-CI score, median (IQR)	1 (2)*	0 (2)	1 (2)	2 (2)	2 (2)	2 (3)
Charlson-CI = 0, *n* (%)	68 (39)^#^	45 (52)	8 (36)	10 (22)	3 (19)	2 (40)
Charlson-CI = 1, *n* (%)	38 (22)^#^	19 (22)	6 (27)	11 (24)	2 (13)	0 (0)
Charlson-CI ≥ 2, *n* (%)	69 (39)^#^	22 (26)	8 (36)	25 (54)	11 (69)	3 (60)
Premorbid Barthel Index, median (IQR)	20 (3)	20 (2)	20 (2)	19 (3)	17 (7)	18 (3)
LoS acute hospital in days, median (IQR)	19 (14)	19 (11)	19 (13)	19.5 (18)	22 (18)	21 (21)
Barthel Index on admission, median (IQR)	12 (10)*	14 (7)	9 (12)	9 (8)	8 (9)	10 (6)
*Variables at discharge*						
LoS rehabilitation in weeks, median (IQR)	12 (15)*	8 (6)	16 (23)	22 (26)	–	16 (6)
Barthel Index at discharge, median (IQR)	17 (8)*	18 (4)	16 (9)	11 (10)	–	15 (4)
Relative functional gain, median (IQR)	67 (90)*	85 (84)	67 (76)	24 (79)	–	71 (42)
Discharge home, *n* (%)	88 (56)^#^	62 (73)	9 (43)	13 (28)	–	4 (80)

Equal statistical significance was found when deceased patients were excluded

*SD* standard deviation, *Charlson-CI* Charlson comorbidity index, *IQR* interquartile range, *LoS* length of stay

Statistical significance at *p* < 0.05

*Kruskal–Wallis test

^#^Chi-Square test

Of the 89 (51%) patients that developed an intercurrent disease, 49% developed one disease, 33% ≥ 2 diseases, and 18% died. Comorbidity was present in 116 (62%) patients: 40 (21%) scored 1 and 76 (41%) scored ≥ 2. The most prevalent pre-existing comorbidities were myocardial infarction (18%), diabetes mellitus (18%) and congestive heart failure (16%).

### Characteristics related to intercurrent diseases

Patients without any intercurrent disease had a BI on admission of at least 4 points higher than those with intercurrent diseases. The proportion of patients without comorbidity was largest in the category ‘no intercurrent disease’ (52%), whereas in the category ‘With impact’, the proportion of patients with multiple comorbidities was the largest (54%), *p* = 0.007. Patients that developed intercurrent diseases were less often discharged home, had a longer LoS, a lower BI at discharge, and a lower relative functional gain. This also applied to the category that was considered as having ‘No impact’. Multivariate analyses showed that: BI on admission (OR 0.87 [0.82–0.92]) and comorbidity (OR 1.43 [1.13–1.81]) were independently associated with the presence of intercurrent diseases, but only the Charlson-CI was significantly associated with number of intercurrent diseases (incidence rate ratio: 1.14 [1.03–1.25], *p*: 0.008). This means that with every extra point on the Charlson-CI, a 14% increase in the number of intercurrent diseases is expected (Supplement material Appendix D).

### Comorbidity and intercurrent diseases

Having diabetes and/or kidney disease was significantly related to the occurrence of an intercurrent disease (Table 2). Moreover, logistic regression analysis showed that only diabetes was independently associated (OR 3.50 [1.32–9.26]). No clear patterns or relations between comorbidities and specific intercurrent diseases were observed: a wide variety of different diseases occurred in patients with pre-existing comorbidity. The intercurrent diseases that most frequently occurred were cardiovascular (13%), psychiatric (12%) such as depression and delirium, and genitourinary (11%), predominantly urinary tract infections. An overview of intercurrent diseases, per comorbidity (the five most prevalent only), is presented in Supplement material Appendix E.

**Table 2:** Associations between comorbid conditions and presence of ≥ 1 intercurrent disease (ID)

Comorbidity	Total	ID absent	ID present
Charlson index, median (IQR)	1 (2)	0 (2)^#^	2 (3)^#^
*Comorbid condition*			
Myocardial infarction, *n* (%)	31 (18)	13	18
Heart failure, *n* (%)	29 (17)	10*	19*
Peripheral vascular disease, *n* (%)	23 (13)	9	14
Dementia, *n* (%)	1 (1)	1	0
Chronic pulmonary disease, *n* (%)	18 (10)	8	10
Musculoskeletal/connective tissue, *n* (%)	9 (5)	2*	7*
Ulcers, *n* (%)	8 (5)	2	6
Mild liver disease, *n* (%)	3 (2)	1	2
Kidney disease (moderate), *n* (%)	16 (9)	**3** ^**#**^	**13** ^**#**^
Diabetes mellitus, *n* (%)	31 (18)	**9** ^**#**^	**22** ^**#**^
Malignancy, *n* (%)	10 (6)	3	7
Leukaemia, *n* (%)	1 (1)	1	0
Lymphoma, *n* (%)	2 (1)	0	2
Moderate liver disease, *n* (%)	0 (0)	0	0
Metastasis of solid tumour, *n* (%)	3 (2)	1	2
Any malignancy (of the above mentioned), *n* (%)	13 (7)	4	9

Comorbidities included in the logistic regression analysis are presented in bold

Chi Square test: * *p* < 0.10, ^#^ *p* < 0.05

### Comorbidity and baseline functional status

Table 3 shows the cumulative effect of the combination of a lower functional status on admission (BI ≤ 14) and the presence of comorbidity (Charlson-CI ≥ 1) in relation to the occurrence of an intercurrent disease. On admission, when comorbidity and lower functional status on admission were present separately, ORs were 1.73 [0.52–5.72] and 1.62 [0.53–4.94], respectively. However, if both were present, the OR was 6.70 [2.33–19.2].

**Table 3:** Comorbidity and baseline function: the separate and combined effect on developing an intercurrent disease in geriatric stroke rehabilitation (*n* = 170)

Charlson-CI score ≥ 1	BI ≤ 14 on admission*	Intercurrent disease		Odds ratio [95% CI]
		Yes	No	
No	No	6	17	Reference 1.00
No	Yes	16	28	1.62 [0.53–4.94]
Yes	No	11	18	1.73 [0.52–5.72]
Yes	Yes	52	22	6.70 [2.33–19.2]

*Charlson-CI* charlson comorbidity index, *BI* Barthel index; *CI* confidence interval

*Assessing the BI on admission was not possible in 5 patients. Sensitivity analysis showed similar results: when deceased patients were excluded (*n* = 154) ORs were 1.32 [0.42–4.11], 1.42 [0.42–4.83] and 5.54 [1.91–16.0], respectively

## Discussion

### Main findings

To our knowledge, this is the first study to focus on comorbidity and intercurrent diseases during geriatric stroke rehabilitation. The study cohort was characterised by a large drop in functional status after acute stroke, often with multiple comorbidities and a higher age compared to the majority of studies on stroke patients [5–10, 12–14, 26]. Although this subgroup had been triaged for inpatient geriatric rehabilitation, and selected as a vulnerable subgroup of patients on the base of medical complexity and functional dependency, discriminant factors were still present. Lower baseline functional status, higher pre-existing comorbidity burden in general and specifically the presence of diabetes mellitus were independent determinants of developing intercurrent diseases. Furthermore, patients with multiple comorbidities (higher Charlson-CI) had an increased risk to develop a higher number of intercurrent diseases. Finally, the odds of developing an intercurrent disease were substantially higher if a patient had both comorbidity and functional impairment than if only one of these factors were present.

### Intercurrent diseases

The percentage of patients (51%) that developed intercurrent diseases is comparable to that of studies using an assessment method similar to ours (i.e. 30–54%) [5, 8, 9, 13, 14, 17]. However, although other studies found a higher rate (60–100%), there was a clear difference in the methods used. For example, shoulder pain, limb spasticity, dysphagia or aphasia were categorised as a complication, whereas in the present study (and similar studies) these were considered to be symptoms and not diseases [6, 7, 10–12, 26]. In this study, we were specifically interested in intercurrent diseases that occurred during the inpatient rehabilitation period, and physicians retrospectively registered the intercurrent diseases. Nevertheless, our incidence rates were similar to those in studies using prospective assessment and similar prevalent diseases were found, i.e. genitourinary (urinary tract infections) and psychiatric diseases (depression and delirium) [6–12, 14, 17]. However, in the present study intercurrent cardiovascular disease was more prevalent, presumably because pre-existing cardiovascular comorbidities were highly prevalent in our subgroup of vulnerable geriatric patients.

### Intercurrent diseases and their associations

The presence of intercurrent diseases was related to rehabilitation impact indices (longer LoS, less functional recovery and less often being discharged home). Despite that physicians retrospectively registered intercurrent diseases according to their influence on rehabilitation, it was striking that this relation also applied to the category ‘No impact’. This underlines the impact that intercurrent diseases can have on rehabilitation outcomes. Besides baseline functional status and comorbidity in general, diabetes mellitus was found to be a significant determinant of the occurrence of an intercurrent disease. Diabetes affects various organ systems (e.g. vascular, skin, eyes, nervous system) and might be the (underlying) cause of a variety of intercurrent diseases. However, the present study had insufficient power to further investigate different comorbidities and their associations with specific intercurrent diseases.

### Comorbidity and functional impairment

The last aim was to focus on comorbidity and functional impairment, as both seem to play an important role in relation to the occurrence of intercurrent diseases. Moreover, our results suggest that the combination of these factors increases the risk of developing intercurrent diseases, even more than would be expected (i.e. the ORs from the separate factors multiplied or summed up). This may indicate that the evaluation of comorbidity and functional status should be integrated, preferably taking into account the functional severity of each comorbid condition. It should be noted that some ORs were not significant due to the small size of the subgroups. A larger study is needed to further investigate this combined effect on developing intercurrent diseases during rehabilitation.

### Strengths and limitations

The strength of the GRAMPS study is its multidisciplinary and multicenter prospective design in a relatively large study population. Whereas most studies on stroke rehabilitation investigated mainly younger patients, the present study represents the older, geriatric stroke patient population relatively well and, therefore, strengthens external validity [27]. The study investigated two outcomes: presence and number of intercurrent diseases. Diseases were recorded using the ICD-10 coding system, and only diseases were scored (i.e. not symptoms such as pain or dysphagia). We believe this prevents confusion regarding definitions and elucidates the role of functional activities (functional status), medical health conditions (comorbidity and intercurrent diseases) and their interactions in the complex setting of rehabilitation and recovery, using the ICF model as a framework [28].

Another strength is the use of a Poisson regression that allowed to analyse the ‘number of intercurrent diseases’. Furthermore, we presented the classifications ‘No impact’ and ‘With impact’. The intercurrent diseases found in this study might be a selection of the more severe diseases, due to the retrospective design of registering the diseases; however, analysing the impact classification as separate groups provided extra information and insight.

Some limitations of the study need to be considered. This study can be considered a secondary analysis, because the GRAMPS study sample size (power) estimation was originally based on the dichotomous outcome measure ‘home discharge’, and a minimum group size of 70 was considered to be appropriate (15). However, in the present study, the groups with and without intercurrent disease were of sufficient size (*n* = 89 and *n* = 86, respectively). Furthermore, the cohort was a specific subgroup of older and vulnerable stroke patients as presented in Supplement material Appendix0020A, and data collection for the GRAMPS study ended some years ago (in 2010). The mean LoS in this study was longer (i.e. ± 4 weeks) compared with recent clinical practice in similar SNFs. Nevertheless, we believe that these data reflect the current situation of geriatric stroke rehabilitation well enough, since no important changes regarding comorbidities or intercurrent diseases are expected.

Finally, comorbidity was assessed using the Charlson-CI in relation to outcomes other than mortality, although the index was specifically designed to predict mortality. Nevertheless, all detected relations showed similar results after performing sensitivity analyses in which deceased patients were excluded.

## Conclusions

Intercurrent diseases frequently occur during geriatric stroke rehabilitation and have a detrimental effect on rehabilitation outcome, such as functional recovery and length of stay. The present study emphasises that comorbidity and functional status need to be integrated and are important factors associated with intercurrent diseases. In particular, diabetes mellitus showed a strong independent association; therefore, this should be a focus for screening, early detection of dysregulation and treatment, to target prevention of various intercurrent diseases. The impact of specific comorbidities and the usefulness of routinely assessing comorbidity combined with integrated functional severity should be further investigated.

## Electronic supplementary material

Below is the link to the electronic supplementary material.

Supplementary material 1 (DOCX 37 kb)

Supplementary material 2 (DOCX 29 kb)

Supplementary material 3 (DOCX 15 kb)

Supplementary material 4 (DOCX 15 kb)

Supplementary material 5 (DOCX 17 kb)
