# Supplementary material for: Comorbidity and intercurrent diseases in geriatric stroke rehabilitation: a multicentre observational study in skilled nursing facilities
Source: Eur Geriatr Med. 2018 Mar 13;9(3):347–53. doi: 10.1007/s41999-018-0043-5 (PMC5972181; doi:10.1007/s41999-018-0043-5)
Supplement: Supplementary file 1 — Supplementary material 1 (DOCX 37 kb) [file 41999_2018_43_MOESM1_ESM.docx]

**Appendix A. Flow chart: characteristics of the Dutch stroke population** (2008-2012)([2](#_ENREF_2))

Dutch population

Note: characteristics of the subgroup in the highlighted frame **(bold)** are similar in the present study. Abbreviations: LoS, length of stay.

Stroke incidence around 40,000 per year

82% living independently at home

11% living at home with extra care

7% other (care home/ in hospital)

Average LoS in acute hospital: 9.5 days

**10%** in hospital mortality

Patients discharged home, independently without any extra care =

**29%**

LoS acute hospital ≈ 5 days

4^th^ day Barthel index usually >15

Age usually < 70 years

Other discharge destinations =

**5%** speciality inpatient neuro rehabilitation

**3%** care home

**1%** nursing home

**1%** unknown

Patients discharged to a skilled nursing facility =

**28%**

LoS acute hospital ≈ 15 days

4^th^ day Barthel index usually <13

Age usually >72 years

Patients discharged home, independently with extra home care =

**23%**

LoS acute hospital ≈ 7 days

4^th^ day Barthel index usually >13

Age usually < 72 years
