# Supplementary material for: Comorbidity and intercurrent diseases in geriatric stroke rehabilitation: a multicentre observational study in skilled nursing facilities
Source: Eur Geriatr Med. 2018 Mar 13;9(3):347–53. doi: 10.1007/s41999-018-0043-5 (PMC5972181; doi:10.1007/s41999-018-0043-5)
Supplement: Supplementary file 2 — Supplementary material 2 (DOCX 29 kb) [file 41999_2018_43_MOESM2_ESM.docx]

**Appendix B. Flow Diagram of the present study cohort**

Patients assessed for eligibility

(n = 378)

Excluded (n = 192)

- Refused study participation (n = 73)
- Critical illness (n = 13)
- Legal incapacity (n = 64)
- Expected short stay (n = 7)
- Randomly not invited to participate, due to logistics (n = 35)

Included in the GRAMPS study

(n = 186)

Lost to follow up (n = 11)

- Translocation (preference of patient) to another facility (n = 5)
- Premature discharge (preference of patient) (n = 3)
- Unknown (n = 3)

Analysed in the present study

(n = 175)
