# Supplementary material for: Comorbidity and intercurrent diseases in geriatric stroke rehabilitation: a multicentre observational study in skilled nursing facilities
Source: Eur Geriatr Med. 2018 Mar 13;9(3):347–53. doi: 10.1007/s41999-018-0043-5 (PMC5972181; doi:10.1007/s41999-018-0043-5)
Supplement: Supplementary file 3 — Supplementary material 3 (DOCX 15 kb) [file 41999_2018_43_MOESM3_ESM.docx]

**Appendix C. Characteristics of the patients that were lost to follow up**

|  | **Missing (n=11)** | **Cohort (n=175)** |
| --- | --- | --- |
| **Age in years, mean (SD)** | 74.8 (10.8) | 78.8 (8.0) |
| **Gender, male (n, %)** | 5 (46) | 79 (46) |
| **LoS in acute hospital, median (IQR)** | 18 (9) | 19 (13) |
| **Independent living before event (n, %)** | 11 (100) | 153 (89) |
| **Charlson-CI score, mean (SD)** | 2.0 (1.8) | 1.5 (1.8) |
| **BI premorbid, median (IQR)** | 20 (2) | 20 (3) |
| **BI on admission, median (IQR)** | 3 (9) | 12 (10) |

Abbreviations: SD, standard deviation; IQR, Interquartile range; LoS, length of stay; Charlson-CI, Charlson comorbidity index; BI, Barthel index.
