# Supplementary material for: Comorbidity and intercurrent diseases in geriatric stroke rehabilitation: a multicentre observational study in skilled nursing facilities
Source: Eur Geriatr Med. 2018 Mar 13;9(3):347–53. doi: 10.1007/s41999-018-0043-5 (PMC5972181; doi:10.1007/s41999-018-0043-5)
Supplement: Supplementary file 4 — Supplementary material 4 (DOCX 15 kb) [file 41999_2018_43_MOESM4_ESM.docx]

**Appendix D. Characteristics independently associated with intercurrent diseases during geriatric stroke rehabilitation.**

| **Variable** | **Presence of ID OR [95% CI]*** | **Number of ID IRR [95% CI]**^#^ |
| --- | --- | --- |
| **Age** | 1.01 [0.97 – 1.05] | 1.01 [0.98 – 1.04] |
| **Gender (male)** | 0.70 [0.36 – 1.39] | 1.19 [0.78 – 1.82] |
| **Barthel index on admission** | 0.87^c^ [0.82 – 0.92] | 0.99 [0.96 – 1.03] |
| **Charlson comorbidity index** | 1.43^a^ [1.13 – 1.81] | 1.14^b^ [1.03 – 1.25] |

Abbreviations: ID, intercurrent disease; OR, odds ratio; CI, confidence interval; IRR, incident rate ratio.

Note: * logistic regression analysis; ^#^ Poisson regression analysis. Statistical significance: ^a^ p< 0.05; ^b^ p< 0.01.; ^c^ p< 0.001. Equal statistical significance when deceased patients were excluded.
