# Supplementary material for: Comorbidity and intercurrent diseases in geriatric stroke rehabilitation: a multicentre observational study in skilled nursing facilities
Source: Eur Geriatr Med. 2018 Mar 13;9(3):347–53. doi: 10.1007/s41999-018-0043-5 (PMC5972181; doi:10.1007/s41999-018-0043-5)
Supplement: Supplementary file 5 — Supplementary material 5 (DOCX 17 kb) [file 41999_2018_43_MOESM5_ESM.docx]

**Appendix E. Number of intercurrent diseases and their impact on rehabilitation goals or length of stay.**

| **ICD 10 code** | **Intercurrent Disease** | **Total**  **n = 175** | **MI and ID**  **n = 18** | **HF and ID**  **n = 19** | **PVD and ID**  **n = 14** | **KD and ID**  **n = 13** | **DM and ID**  **n = 22** | **Deceased**  **n = 16** |
| --- | --- | --- | --- | --- | --- | --- | --- | --- |
| I A00-B99 | Generalised infection | 6 | 0 | 3 | 0 | 0 | 2 | 2 |
| II C00-D48 | Neoplasm | 4 | 1 | 1 | 1 | 1 | 1 | 0 |
| III D50-D89 | Haematological | 3 | 0 | 2 | 1 | 3 | 0 | 0 |
| IV E00-E90 | Endocrine | 4 | 0 | 1 | 1 | 0 | 1 | 0 |
| V F00-F99 | Psychiatric/ delirium | 21 | 3 | 4 | 2 | 2 | 2 | 0 |
| VI G00-G90 | Neurological | 8 | 2 | 1 | 2 | 0 | 2 | 5 |
| VII H00-H59 | Ocular | 4 | 1 | 1 | 1 | 1 | 1 | 0 |
| VIII H60-H95 | Ear/nose/throat | 0 | 0 | 0 | 0 | 0 | 0 | 0 |
| IX I00-I99 | Cardiovascular | 22 | 4 | 7 | 4 | 2 | 4 | 6 |
| X J00-J99 | Pulmonary | 15 | 2 | 3 | 0 | 1 | 2 | 3 |
| XI K00-K93 | Gastrointestinal | 14 | 1 | 2 | 3 | 2 | 2 | 0 |
| XII L00-L99 | Dermatological | 5 | 0 | 1 | 0 | 2 | 2 | 0 |
| XIII M00-M99 | Musculoskeletal | 9 | 1 | 2 | 2 | 2 | 3 | 0 |
| XIV N00-N99 | Genitourinary | 19 | 9 | 5 | 4 | 6 | 6 | 0 |
| XVIII R00-R99 | Not otherwise specified | 4 | 2 | 0 | 2 | 1 | 3 | 0 |
| XIX S00-T98 | Iatrogenic injury or intoxication | 1 | 0 | 0 | 0 | 0 | 0 | 0 |

Abbreviations: ICD, International Classification of Diseases; ID, intercurrent disease; MI, myocardial infarction; HF, heart failure; PVD, peripheral vascular disease; KD, kidney disease; DM, diabetes mellitus.

Note: a patient could have multiple comorbidities and intercurrent diseases.
